# Fontan Surgery in a Middle-Income Country: Outcomes and Modifiable Factors Potentially Associated with Risk - A 13-Year Single-Center Experience

**DOI:** 10.21470/1678-9741-2024-0238

**Published:** 2026-05-06

**Authors:** Renata Medina dos Santos, Marcelo Goulart Correia, Andrey José de Oliveira Monteiro, Luiz Fernando Canêo, Denoel Marcelino de Oliveira, Cristiane da Cruz Lamas

**Affiliations:** 1 Division of Research, Instituto Nacional de Cardiologia, Rio de Janeiro, Rio de Janeiro, Brazil; 2 Division of Statistics and Informatics, Instituto Nacional de Cardiologia, Rio de Janeiro, Rio de Janeiro, Brazil; 3 Division of Surgical Procedures, Instituto Nacional de Cardiologia, Rio de Janeiro, Rio de Janeiro, Brazil; 4 Pediatric Cardiovascular Surgery Service, Department of Cardiovascular Surgery, Instituto do Coração do Hospital das Clínicas da Faculdade de Medicina da Universidade de São Paulo (InCor-HC-FMUSP), São Paulo, São Paulo, Brazil; 5 Instituto Nacional de Infectologia Evandro Chagas, Fiocruz, Rio de Janeiro, Rio de Janeiro, Brazil

**Keywords:** Fontan Procedure, Mortality, Congenital Heart Defects, Univentricular Heart, Brazil.

## Abstract

**Introduction:**

Functionally univentricular hearts represent nearly 10% of all congenital heart defects, and the Fontan procedure is used in the heterogeneous group of patients who have them. Although early mortality after the Fontan procedure has declined internationally, data from middle-income countries remain scarce.

**Methods:**

This is a single-center retrospective observational study made through a review of the medical records of all pediatric patients who underwent the Fontan procedure from 2007 to 2020 in a public cardiac surgery referral center in Brazil.

**Results:**

Fontan procedure with an extracardiac conduit was performed in 78 children (52.6% female), predominantly for tricuspid atresia (55.1%), at a mean age of 12.1 ± 3.2 years. Preoperative oxygen saturation averaged 78.6 (± 7.4)%. Thirty-day mortality was 13.0%. Mortality decreased from 22.9% before 2015 to 4.8% after 2015 (P = 0.037), concurrent with yearly increase in surgical volume and a rise in operating-room extubation rates (from 2.9% to 47.6%, P = 0.001). One in five patients presented with malnutrition or low body weight. In the multivariate analysis, we found a hazard ratio of 65.05 (95% confidence interval: 7.923 - 534.1; P = 0.0001) for in-hospital mortality associated with postoperative acute kidney injury.

**Conclusions:**

Patients underwent the Fontan procedure at a relatively late age. Mortality decreased over time alongside increased surgical volume and operating-room extubation rates. Nutritional status emerged as a potentially modifiable risk factor, whereas postoperative acute kidney injury was a strong independent predictor of in-hospital mortality, underscoring the critical impact of perioperative care.

## INTRODUCTION

**Table:** 

Abbreviations, Acronyms & Symbols				
AKI	= Acute kidney injury		DORV	= Double outlet right ventricle
AV	= Atrioventricular		HLHS	= Hypoplastic left heart syndrome
AVSD	= Atrioventricular septal defect		HR	= Hazard ratio
AVV	= Atrioventricular valve		ICU	= Intensive care unit
BARC	= Bleeding Academic Research Consortium		INR	= International normalized ratio
BMI	= Body mass index		mPAP	= Mean pulmonary artery pressure
BTT	= Blalock-Thomas-Taussig		NYHA	= New York Heart Association
CCTGA	= Congenitally corrected transposition of the great arteries		PAB PTFE	= Pulmonary artery banding = Polytetrafluorethylene
CI	= Confidence interval		RV	= Right ventricle
CPB	= Cardiopulmonary bypass		SatO₂	= Oxygen saturation
DILV	= Double inlet left ventricle		SD	= Standard deviation
DIRV	= Double inlet right ventricle		TGA	= Transposition of the great arteries

The Fontan procedure is an eponym for total cavopulmonary connection. Fontan and Baudet reported two successful cases in 1971, initially by means of atriopulmonary anastomosis, both patients having tricuspid atresia^[1]^.

When mentioning the “Fontan” concept, Gewillig and Brown state that the normal cardiovascular system consists of two synchronized pumps that drive blood to the pulmonary and systemic bed, while in the Fontan circulation there is no propelling pump in the pulmonary artery, since the venous system is directly connected to the pulmonary artery^[2]^. Functionally univentricular hearts represent approximately 10% of all congenital heart defects^[3]^.

Patients undergoing the Fontan procedure are a heterogeneous group, with different underlying congenital heart diseases, and most have undergone previous procedures, mainly the bidirectional cavopulmonary anastomosis (Glenn procedure), performed through the same access route, the median sternotomy. Such characteristics bring complexity and risk to the surgery. Over the years, the chronicity of venous system hypertension and suboptimal cardiac output eventually lead to end-organ dysfunction^[4]^.

The systematic review by Kverneland, Kramer, and Ovroutski (1971 - 2016), including over 40 centers, showed a clear decline in early mortality and a trend toward improved long-term survival after the Fontan procedure. No centers from Central or South America or Africa were included, highlighting that efforts to improve postoperative management and reduce early and late morbidity have largely occurred within international community without the representation from these regions^[5]^.

Caneo et al.^[6]^ reported an overall early mortality of 11% in 420 patients who underwent the Fontan procedure between 1984 and 2014 at a Brazilian center. When stratified into three eras, early mortality (deaths within 30 days of surgery or during the same hospitalization) was 6.5%, 4.7%, and 9.3%, respectively. Although the difference was not statistically significant, the authors suggested that the increase in mortality may have resulted from inadequate patient selection and broadened surgical indications.

There is a wide spectrum of clinical outcomes after the Fontan procedure in the different centers^[2]^. The institution where the procedure is performed is a strong independent predictor of length of hospital stay after the Fontan procedure, although it was not possible to determine which practices in the different centers were the cause of these results^[7]^.

Knowledge of the reality of each center is relevant, especially in the management of congenital heart diseases, due to the complexity, rarity, and singularity of the cases. Thus, the objective of this study is to describe a series of cases of pediatric patients who underwent the Fontan procedure at a federal public Brazilian hospital, from 2007 to 2020, presenting the profile of these patients and assessing mortality and associated risk factors over the period. Our primary objective is to report institutional outcomes from a middle-income country - a setting that remains underrepresented - thereby contributing to reducing the geographic gap in knowledge about the Fontan procedure, with special emphasis on factors associated with early mortality and hospital readmissions.

## METHODS

### Study Type and Place

This is a single-center, retrospective observational study. The center is a federal public hospital located in the city of Rio de Janeiro, Brazil. It is recognized by the Brazilian Ministry of Health as a reference center for the treatment of complex heart diseases.

### Inclusion and Exclusion Criteria

All patients who underwent the Fontan procedure at a reference hospital for complex congenital heart diseases in Brazil, between August 2007 and July 2020, were included. Patients under 18 years of age (pediatric patients) at the time of total cavopulmonary connection were identified by the hospital management department lists.

Any patient who did not meet the inclusion criteria was excluded.

### Ethics

This study was approved by the Institutional Scientific Committee in September 2020 and by the Research Ethics Committee, under the number: 37703420.3.0000.5272. Informed consent was waived because it was a retrospective observational study with review of medical records.

### Data Collection

The database was created by consulting the medical records of the patients and the Portal Extrajudicial - Consulta de Nascimentos e Óbitos of the Judiciary Power of Rio de Janeiro, Brazil.

Data were collected through the electronic data capture tool for research REDCap (Research Electronic Data Capture), version 12.5.5, hosted at the hospital.

Data on variables of preoperative, intraoperative, and postoperative periods were collected from physical and electronic medical records. All collected variables were considered present only if documented in the patients' medical records, including liver failure, acute kidney injury (AKI), postoperative dialysis, seizures, stroke, and neurological events (excluding seizures and stroke).

The mean pulmonary artery pressure (mPAP) was obtained from the catheterization before the procedure. Atrioventricular valve dysfunction was obtained from the echocardiogram before the procedure. Drainage time was defined as the interval between surgery and the removal of all chest tubes; prolonged drainage time was defined as a duration > 10 days; prolonged cardiopulmonary bypass (CPB) time as duration > 120 minutes; prolonged intubation as mechanical ventilation lasting more than 24 hours; and prolonged hospital stay as a duration exceeding 14 days, calculated from the day of admission to the day of hospital discharge.

The perioperative results associated with morbidity and mortality within 30 days and up to hospital discharge of patients undergoing the Fontan procedure were described. Results were divided into periods before (and including) 2015 and after 2015, for comparison of periods with similar numbers of patients, aiming to evaluate changes in outcomes over time.

Only readmissions to the study center were evaluated. The causes of readmission were classified as related to the Fontan procedure or the underlying congenital heart disease, or as unrelated.

### Statistical Analysis

For the statistical analysis and graphs, the Jamovi project program, version 2.3, using the statistical language R, version 4.1, was used. The significance level adopted was 5%.

Descriptive data analysis was performed. Continuous numeric variables were presented as median and interquartile range (25^th^ and 75^th^ percentiles), or mean and standard deviation (SD), depending on their non-normal or normal distribution, respectively. Categorical variables were presented in frequencies and percentages. The associations were calculated using the Student's *t*-test or the Mann-Whitney U test, according to normality or not, respectively, after testing normality using the Shapiro-Wilk test. Categorical variables were compared using the Chi-square and Fisher's exact tests. The *t*-test for independent samples was used.

A subgroup analysis was conducted to compare patients who underwent the Fontan procedure before and after 2015, assessing baseline characteristics and a range of in-hospital postoperative events.

A Cox regression analysis for mortality was performed. The Kaplan-Meier curve was generated, showing readmission-free survival.

## RESULTS

There were 78 Fontan procedures in the study period. There was a predominance of females (52.6%), and mean patient age at the time of the Fontan procedure was 12.1 (± 3.2) years. On the echocardiogram prior to the Fontan procedure, the predominant baseline diagnosis was tricuspid valve atresia, present in 43/78 (55.1%) of the patients. Characteristics of the population of children undergoing the Fontan procedure are shown in Table 1.

**Table 1:** Characteristics of the population of children undergoing Fontan procedure (N = 78), Rio de Janeiro, RJ, Brazil, 2007 - 2020.

Characteristics	n/N	%	Mean^a^/Median^b^
Age at Fontan (years)	78/78		12.1 (± 3.2)
Female	41/78	52.6 %	
Weight (Kg)	78/78		35.5 (± 12.1)
BMI (kg/m^2^)	57/78		16 (14.4, 18.1)
BMI (*z*-score)			
Severe malnutrition	5/57	8.8%	
Low weight	7/57	12.3%	
Eutrophic	32/57	56.1%	
Overweight	8/57	14.0%	
Severe obesity	5/57	8.8%	
Pre-Fontan functional class	56/78		
NYHA I	9/56	16.1%	
NYHA II and III	46/56	82.1	
NYHA IV	01/56	1.8%	
Previous surgery	58/78	74.3%	
Modified BTT	48/78	61.5%	
PAB	10/78	12.8%	
mPAP (mmHg)	76/78		12.7 (± 3.3)
mPAP (> 15 mmHg)	22/78	28.9%	0.6 (± 0.1)
Age at Glenn surgery (years)	78/78		2.3 (1.6; 5.0)
Pre-Fontan hematocrit (%)	78/78		56.9 (± 5.0)
Pre-Fontan SatO₂ (%)	76/78		78.6 (± 7.4)
Pre-Fontan creatinine (mg/dL)	78/78		0.6 (± 0.1)
Main ventricle type	76/78		
Left ventricle	64/76	82.1%	
Right ventricle	5/76	6.4%	
Biventricular	08/76	10.3%	
Undetermined	01/76	1.3%	
Ejection fraction (%)	40/78		59.8 (± 8.6)
Function of the main ventricle			
Preserved	64/76	84.2%	
Mild dysfunction	11/76	14.5%	
Moderate dysfunction	01/76	1.3%	
AVV regurgitation			
No regurgitation or mild	59/63	93.6%	
Moderate or severe	4/63	6.4%	
Base morphology			
Tricuspid valve atresia	43/78	55.1 %	
Pulmonary stenosis	34/78	43.6%	
Pulmonary atresia	21/78	26.9%	
DILV	12/78	15.4%	
DORV	10/78	12.8%	
Dextrocardia	10/78	12.8%	
Single AVV	7/78	9.0%	
Right isomerism	5/78	6.4%	
CCTGA	3/78	3.9%	
TGA	2/78	2.6%	
Tricuspid valve hypoplasia	2/78	2.6%	
Mitral atresia	2/78	2.6%	
DIRV	1/78	1.3%	
Total AVSD	1/78	1.3%	
HLHS	1/78	1.3%	

a mean values and standard deviations;

b median with interquartile range

AVSD=atrioventricular septal defect; AVV=atrioventricular valve; BMI=body mass index; BTT=Blalock-Thomas-Taussig; CCTGA=congenitally corrected transposition of the great arteries; DILV=double inlet left ventricle; DIRV=double inlet right ventricle; DORV=double outlet right ventricle; HLHS=hypoplastic left heart syndrome; mPAP=mean pulmonary artery pressure; NYHA=New York Heart Association; PAB=pulmonary artery banding; SatO2=oxygen saturation; TGA=transposition of the great arteries

Most patients (74/78; 94.9%) were residents of the state of Rio de Janeiro, Brazil. The median declared family income was 1.8 (1.2, 2.6) minimum wages, the lowest income was zero and the highest was ten minimum wages. The per capita family income in minimum wages was 0.4 (0.27, 0.65). The predominant level of education that the children’s guardians declared was incomplete primary education in 34.4% of fathers and 44.3% of mothers.

Intraoperative characteristics of children who underwent the Fontan procedure are described in Table 2. All patients underwent the Fontan procedure with an extracardiac conduit, except one with missing data.

**Table 2:** Intraoperative characteristics of children undergoing Fontan procedure (N = 78), Rio de Janeiro, RJ, Brazil, 2007 - 2020.

Feature	n/N	%	Median^a^
Fontan type: extracardiac conduit	77/77	100%	
PTFE conduit size (mm)	77/77		
16 mm	8	10.4%	
18 mm	23	29.9%	
20 mm	40	51.9%	
22 mm	6	7.8%	
CPB time (minutes)	77/78		75 (60, 93)
Prolonged CPB time (> 120 minutes)	11/77	14.3%	
Presence of ischemia time	16/77	20.8%	
Ischemia time (clamped aorta)	16/16		19.5 (8.998, 31.250)
CPB time (minutes)	34/77	44.2%	
Prolonged CPB time (> 120 minutes)	12/78	15.4%	
Type of concurrent procedure			
Atrial septostomy	7	58.3%	
Pulmonary artery plasty	4	33.3%	
AV valve repair	3	25.0%	
Subaortic membrane resection	2	16.7%	
Extubation in the operating room (%)	21/77	27.3%	

a median with interquartile range

AV=atrioventricular; CPB=cardiopulmonary bypass; PTFE=polytetrafluorethylene

The intubation time (50/56) was 26.5 (13.2, 50.0) hours; and the drainage time (70/78) was 6.0 (4.0, 11.0) days following the Fontan procedure.

**Fig. 1:**
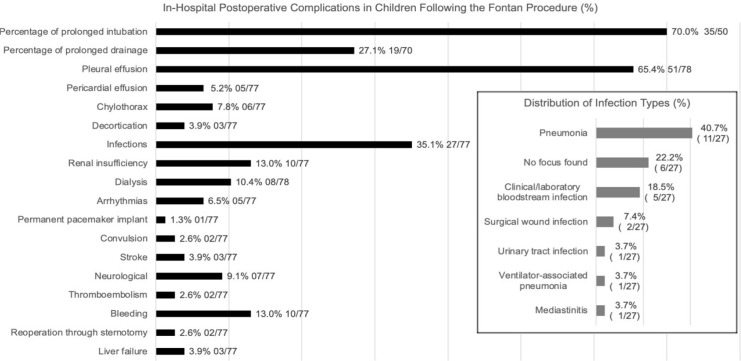
Bar chart showing in-hospital postoperative complications and the distribution of infection types (%) in children following the Fontan procedure, 2007 - 2020.

In-hospital postoperative complications of children following the Fontan procedure are shown in Figure 1: 5/77 (6.5%) patients had arrhythmias, such as sinus tachycardia, and non-sustained atrial tachycardia and atrial flutter; 1/77 (1.3%) patients required permanent pacemaker implantation during the same hospitalization due to total atrioventricular block (this patient had undergone subaortic membrane resection).

Pleural effusion was observed in 51/78 (65.4%) patients during their stay in the intensive care unit (ICU). Chest tube insertion was required in 23/51 (45.1%) patients, on the right side (19/51; 37.3%), followed by the left side (8/51; 15.7%). The median duration of right-sided pleural drainage was 10.0 days (7.5, 15.5), and for left-sided drainage was 6.0 days (3.0, 35.5).

Pericardial effusion occurred in 4/77 (5.2%) patients, and chylothorax was diagnosed in 6/77 (7.8%) patients during the ICU stay. Among patients with chylothorax, 2/6 (33.3%) required chest tube insertion. Surgical decortication was performed in 3/77 (3.9%) patients.

In-hospital postoperative AKI occurred in 10/77 (13.0%) patients, and 8/10 (80%) of them required dialysis. Considering all cases with available data, the need for dialysis was 8/77 (10.4%).

During the postoperative hospital stay, 2/77 (2.6%) patients had a convulsion, 3/77 (3.9%) patients had a stroke, and 7/77 (9.1%) had other neurological complications (excluding convulsion and stroke), which were: absence of trunk reflex, progressing to death, coma with the electroencephalogram showing cerebral electrical silence without clinical signs of brain death, brain death, dysphasia with normal brain computed tomography scan, left brachial plexus injury with neuropathic pain, delirium, and headache.

Bleeding occurred in 10/77 (13.0%) patients, and was manifested as hemothorax in four patients, and in one patient each as gastrointestinal hemorrhage, bleeding from the surgical wound, dyscrasia, gastrointestinal and pulmonary hemorrhages, hemorrhage after decortication requiring a new procedure, and reoperation through sternotomy because of bleeding.

Thromboembolism occurred in 2/77 (2.6%) patients, one of whom had a thrombosis of the Fontan tube, needing dilation and percutaneous stent implantation in the same hospitalization, 15 days after surgery. One patient had an image of a thrombus in the right atrium detected on the transesophageal echocardiogram in the operating room, but which disappeared during surgery; in this patient, a search and dissection of the femoral region was performed to remove the thrombus, but he evolved with significant vasculitis of the lower limbs, dying of cardiogenic shock.

No patient underwent cardiopulmonary support with pediatric extracorporeal membrane oxygenation which became available at the hospital only in 2010.

Infection occurred in 27/77 (35.1%) patients, and pneumonia was the most frequently found, representing 40.7% of the total. The only patient who developed mediastinitis died of sepsis seven days after the operation.

Reoperation through sternotomy was necessary in 2/77 (2.6%) patients, one due to bleeding and clots, and the other due to hematoma. One patient had to keep an open chest at the end of the surgery, which was closed in a second surgery, on the third postoperative day.

The median time from hospital admission to surgery, length of stay in the ICU, and length of postoperative hospital stay were 6.0 (2.3, 9.8), 7.0 (4.0, 12.0), and 17.0 (11.8, 27.0) days, respectively. The median length of total hospital stay was 24.5 (17.8, 36.5) days, and 56/68 (82.4%) patients experienced prolonged hospital stay (> 14 days). Most patients, 63/68 (92.6%), were receiving anticoagulation at hospital discharge.

**Fig. 2:**
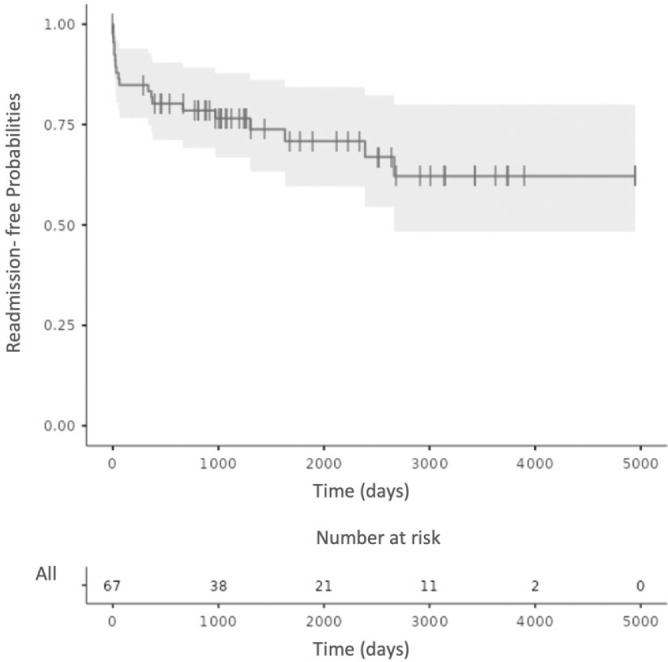
Bar chart showing the annual trends of patients undergoing the Fontan procedure, 2007 - 2020, in-hospital mortality, and operating-room extubation.

Mortality during the study period was 11/77 (14.3%), and ten patients died in-hospital and in less than 30 days (10/77 or 13.0%). Cardiogenic shock was the main cause of death reported in the medical records (4/10 [40%]), followed by septic shock or sepsis (2/10 [20%]), refractory shock (2/10 [20%]), multiple organ dysfunction (1/10 [10%]), and vasoplegic shock (1/10 [10%]). The median time between surgery and death was 4.5 (8.0, 17.5) days. Figure 2 shows yearly increase in surgical volume, decrease in in-hospital mortality, and rise in the number of patients extubated in the operating room.

Follow-up was possible in 67/68 (98.5%) patients who were discharged from hospital and had a median duration of 4.7 years (1721 days), with a minimum of 322 days and a maximum of 13.6 years (4971 days). One patient was discharged after 13 days of hospitalization, was referred back to his state of origin, and was lost to our follow-up after hospital discharge.

In the postoperative follow-up, 1/67 patient (1.5%) required permanent pacemaker implantation less than 30 days after hospital discharge. Bleeding occurred in 7/67 (10.4%) patients, six of which were Bleeding Academic Research Consortium (BARC) 2 and one BARC 3b^[8]^. No patient underwent a Fontan conversion.

Three patients (4.5%) evolved with obstruction of the Fontan tube. Five patients (7.5%) required percutaneous interventions during follow-up: three of them underwent stent implantation in the Fontan tube, one due to obstruction, one due to occlusion of two large caliber aortopulmonary collaterals, and the last one due to occlusion of a pulmonary arteriovenous fistula and the Fontan fenestra.

It is noteworthy that the same patient who underwent pulmonary decortication also evolved with BARC 2 bleeding, obstruction of the Fontan tube with stent placement, and eventually died of septic shock, on day 3,990 of follow-up.

One patient underwent heart transplantation 2,723 days after surgery. This patient was asymptomatic at his last outpatient appointment in February 2022. There were 43/67 (64.2%) patients who were free from any hospital readmission. Readmission in our center, related to congenital heart disease and the Fontan procedure, occurred in 40/51 (78.4%) patients.

**Fig. 3:**
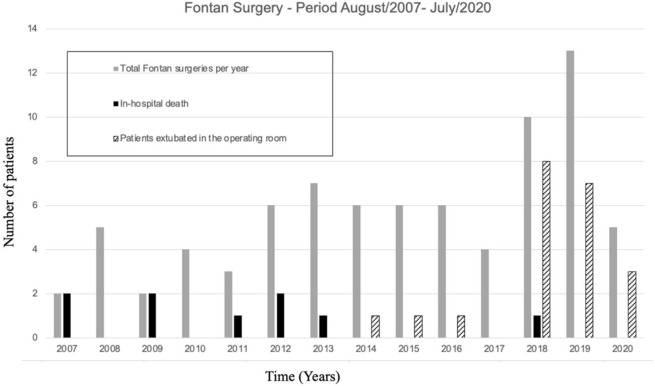
Readmission-free probability curve related to congenital heart disease and surgery over time (in days) in 67 children who had the Fontan procedure, 2007 - 2020.

Figure 3 shows the percentage Kaplan Meier curve for readmission related to congenital heart disease and surgery. The causes of readmission related to congenital heart disease or surgery were several: five patients had bleeding with or without prolonged international normalized ratio (INR), two patients had pleural and/or pericardial effusion, three patients had arrhythmias (one was diagnosed with the Wolff-Parkinson-White syndrome, one with supraventricular electrical instability, and one had symptomatic sinus node dysfunction requiring pacemaker implantation), and one patient had anasarca and prolonged INR. Other complications resulting in readmissions were occlusion of two aortopulmonary collaterals with coil (one patient), heart transplantation with pulmonary artery repair (one patient), therapeutic catheterization for obstruction of the Fontan tube with stent implantation (one patient), occlusion of the arteriovenous fistula and occlusion of the fenestration of the Fontan tube (one patient), rupture of the ascending aorta with cardiac tamponade (one patient), aortic valve endocarditis requiring surgical valve replacement (one patient), and correction of lymphatic fistula in the inguinal region (one patient).

The comparison between patients who underwent fenestration and those who did not showed no difference in prolonged drainage time (*P* = 0.661), as presented in Table 3. However, there was a significant difference between prolonged intubation time and prolonged drainage time (*P* = 0.027), and all patients with a prolonged hospital stay (> 14 days) also had prolonged drainage time, as shown in Table 3.

**Table 3:** Comparison between intraoperative variables and prolonged drainage in children undergoing Fontan procedure (N = 78), Rio de Janeiro, RJ, Brazil, 2007 - 2020.

Variables	Prolonged drainage^a^		
	Yes	No	*P*-value
Main ventricle: RV	2 (10.5%)	3(5.9%)	0.608^b^
Main ventricle: different from RV	17 (89.5%)	48 (94.1%)	
SatO₂ < 75%	4 (22.2%)	12 (24.0%)	1.00^b^
SatO₂ ≥ 75%	14 (77.8%)	38 (76.0%)	
mPAP ≥ 15 mmHg	9 (47.4%)	10 (20.4%)	0.026^c^
mPAP < 15 mmHg	10 (52.6%)	39 (79.6%)	
Prolonged CPB time^d^	3 (15.8%)	7 (13.7%)	1.00^b^
Not prolonged CPB time	16 (84.2%)	44 (86.3%)	
Non-fenestrated Fontan tube	11 (57.9%)	26 (52.0%)	0.661^c^
Fenestrated Fontan tube	8 (42.1%)	24 (48.0%)	
Prolonged intubation time^e^	12(85.7%)	23 (65.7%)	0.294^b^
Non-prolonged intubation time	2 (14.0%)	12 (34.0%)	
Prolonged length of hospital stay^f^	18(100.0%)	37 (75.5%)	0.027^b^
No prolonged length of hospital stay	0 (0.0%)	12 (24.5%)	

a more than ten days;

b Fisher's exact test;

c Chi-square test;

d greater than 120 minutes;

e longer than 24 hours; ^f^longer than 14 days

CPB=cardiopulmonary bypass; mPAP=mean pulmonary artery pressure; RV=right ventricle; SatO₂=oxygen saturation

Prolonged intubation time was present in 31 (93.9%) children who had a long length of hospital stay, as seen on Table 4.

**Table 4:** Comparison between intraoperative variables and prolonged intubation time in children who had the Fontan procedure (N = 78), Rio de Janeiro, RJ, Brazil, 2007 - 2020.

Variables	Prolonged intubation time^a^		
	Yes	No	*P*-value
SatO₂ < 75%	8 (23.5%)	2 (14.3%)	0.701^b^
SatO₂ ≥ to 75%	26 (76.5%)	12 (85.7%)	
mPAP ≥ to 15 mmHg	12 (36.4%)	4 (26.7%)	0.742^b^
mPAP < 15 mmHg	21 (63.6%)	11(73.3%)	
Prolonged CPB time^c^	7 (20.0%)	0 (0.0%)	0.087^b^
Not prolonged CPB time	28(80.0%)	15 (100.0%)	
Non-fenestrated Fontan tube	15 (44.1%)	9 (60.0%)	0.305^d^
Fenestrated Fontan tube	19 (55.9%)	6 (40.0%)	
Prolonged length of hospital stay^e^	31(93.9%)	10 (66.7%)	0.024^b^
No-prolonged length of stay	2 (6.1%)	5 (33.3%)	

a longer than 24 hours;

b Fisher's exact test;

c greater than 120 minutes;

d Chi-square test;

e longer than 14 days

CPB=cardiopulmonary bypass; mPAP=mean pulmonary artery pressure; SatO₂=oxygen saturation

The Cox regression analysis for in-hospital mortality showed a hazard ratio (HR) of 34.4 (95% confidence interval [CI]: 7.056 - 167.7; *P* < 0.0001) for AKI, a HR of 16.8 (95% CI: 4.666 - 60.51; *P* < 0.0001) for the need for dialysis, and HR of 5.829 (95% CI: 1.452 - 23.4; *P* = 0.0129) for neurological events (excluding convulsion and stroke). In the multivariate analysis, we found a HR of 65.05 (95% CI: 7.923 - 534.1; *P* = 0.0001) for postoperative in-hospital AKI.

Considering the period up to and including 2015 and after 2015, there was an increase in the number of annual surgeries, from 4.6 surgeries per year to 6.8 surgeries per year.

A subgroup analysis was performed to compare patients who underwent the Fontan procedure before and after 2015. No significant differences were found between the groups regarding baseline characteristics and a range of in-hospital postoperative events. However, there was a difference in 30-day mortality between patients who had the Fontan procedure up to 2015 (included) and after 2015 (*P* = 0.037), and there was also a difference in the percentage of patients extubated between these two time periods (*P* = 0.001), as shown on Table 5. A higher proportion of patients had postoperative AKI and the need for hemodialysis until 2015.

**Table 5:** Comparison between the variables death, extubation, renal failure, dialysis, and drainage time in the periods up to 2015 and after 2015 in children who had the Fontan procedure (N = 78), Rio de Janeiro, RJ, Brazil, 2007 - 2020.

Variables	Period		
	Until 2015 (inclusive)	After 2015 (exclusive)	*P*-value
Death within 30 days	8 (22.9%)	2 (4.8%)	0.037^a^
Alive within 30 days	27 (77.1%)	40 (95.2%)	
Extubation in the operating room	1 (2.9%)	20 (47.6%)	0.001^a^
No extubation in the operating room	34 (97.1%)	22 (52.4%)	
Postoperative renal failure	8 (22.9%)	2 (4.8%)	0.037^a^
No postoperative renal failure	27 (77.1%)	40 (95.2%)	
Postoperative dialysis	7 (19.4%)	1 (2.4%)	0.021^a^
No postoperative dialysis	29 (80.6%)	41 (97.6%)	
Drainage time (days)^b^	4.0 (7.0, 10.8)	5.0 (4.0, 11.0)	0.858^c^

a Fisher's exact test;

b longest drain time;

c Mann-Whitney U test

## DISCUSSION

Our study contributes to reducing the geographical gap in knowledge about the Fontan procedure in developing countries. It shows that children were operated late, at the age of 12 years, and overall mortality was 14.3%, but it was reduced when comparing the period before and including 2015 and after 2015. In-hospital death was associated with AKI.

### Delayed Age at Fontan

In our cohort, the mean age at the time of the Fontan procedure was relatively high, 12.1 years (SD ± 3.2). These results reflect that late referral for Fontan completion remains frequent in the Brazilian public health system, as reported by Caneo et al.^[6]^, where the median age was eight years^[6]^. In our center, this approach was partly due to the clinical management strategy, which aimed to postpone exposure to surgical risks and to recommend the Fontan procedure typically when clinical evolution made it indispensable, as well as the late referral of patients to our institute.

As a consequence, patients may not fully benefit from the advantages of the staged surgical approach of the Fontan procedure^[6]^, typically performed at younger ages as described in many developed countries: Canada (Mutsuga et al.^[9]^, mean age 3.9 years, SD ± 2.2), Germany (Ovroutski et al.^[10]^, median 3.8 years [1.3 - 37]), and the United States of America (Akintoye et al.^[11]^, median three years).

Akintoye et al.^[12]^ assessed the age-associated risk of in-hospital mortality in patients who had the Fontan procedure using the United States of America’s national inpatient system. They reported that the Fontan procedure is most frequently done in patients aged two years, but the age of three years was the one most associated with the lowest in-hospital mortality. An increased risk of death was seen after three years of age, with an increase in the adjusted risk rates of in-hospital mortality, with values from 2.6% to 8.4% for procedures performed after 10 years.

In our study, patients underwent the Glenn procedure at a median age of 2.3 (1.6; 5.0) years, and the time between the Glenn procedure and the Fontan procedure was 8.6 (± 3.4) years. Ono et al.^[13]^, in a study with 434 patients who had the Fontan procedure between 1994 and 2015 at a single center in Germany, reported that the median age of patients when undergoing partial cavopulmonary connection was 0.5 [0.3-1.1] years, and the interval between this surgery and the Fontan procedure had a median of 1.5 [1.2-2.2] years.

Thus, patients remained for long years with low oxygen saturation. It is noteworthy that the mean oxygen saturation of the patients in our study before the Fontan procedure was 78.6 (± 7.4)%, lower than that published by Mery et al.^[14]^ with 610 patients who had the Fontan procedure at a single center in the United States of America, whose systemic saturation had a mean of 85 (± 5.0)%. These patients, subjected to chronic hypoxemia and reduced pulmonary blood flow, consequently developed increased end-diastolic volume and progressive decline in ejection fraction, which may worsen with advancing age.

### The Importance of Body Composition

Although most patients (56.1%) in our study had normal body mass index (BMI), severe malnutrition was seen in 8.8% of them, and low body weight in 12.3%; when added up, these two categories accounted for 21.1% of patients. Baldini et al.^[15]^ concluded that malnutrition is a problem at any stage of life for a child with a single ventricle, resulting in longer hospital stays, higher mortality, and greater risks of problems in neurodevelopment and growth. Thus, there is an opportunity to improve nutritional parameters modifiable before surgery in our center, since one in five patients presented with severe malnutrition or low body weight.

Anderson et al.^[16]^ concluded that low weight-for-age z-score (< -2) is associated with high rates of postoperative infections in patients who have the Fontan procedure, such as bacteremia, mediastinitis, urinary tract infection, and pneumonia. However, we could not demonstrate an association between low weight and malnutrition with a worse outcome in our study, possibly due to the small numbers of our sample.

BMI values were found in only 73.1% of the patients' charts. Height data of patients during hospitalization for the Fontan procedure were not found in the medical records, while 100% of patients' weights were identified. This issue has been previously highlighted by Baldini et al.^[15]^, who reported that several studies failed to measure anthropometric data throughout the different surgical stages. They emphasized that a thorough assessment of body composition is crucial for understanding nutritional status and for defining more effective nutritional strategies.

Severe obesity was found in 5/57 (8.8%) of the patients, and overweight in 8/57 (14.0%) of the patients, totaling 13/57 (22.8%) patients with obesity and overweight combined. These data suggest that these patients suffer from the same problems as the general population. It is worth mentioning that the American Heart Association in the scientific statement for the evaluation and management of children and adults with the Fontan circulation mentions obesity as a modifiable risk factor for the development of atrial fibrillation and diastolic dysfunction^[17]^. In our study, it was not possible to identify a worse outcome in obese children.

### Socioeconomic Conditions

Regarding socioeconomic conditions, in a multicenter study in the United States of America, with 323 patients submitted to the Fontan procedure, < 17% of patients were classified as below the federal poverty level (minimum income level considered adequate in each country)^[18]^. In our study, 26.5% of patients had a per capita income < US$0.29 (the appropriate income level in Brazil, according to The World Bank, is US$1.9 a day, considering the minimum wage in Brazil as R$1.045,00 and US$1 = R$5,36, both referring to July 2020).

Thus, we observed that there is a high percentage of patients below the poverty level in our study. Additionally, the majority resided far from the city where the study center is located, and incomplete primary education was the most commonly reported level among the children’s guardians.

While it is evident that these patients have access to the public health service, since the hospital provides services entirely through the Brazilian public health system, our findings indicate that their families face significant financial and social barriers to providing adequate support for children undergoing the Fontan procedure.

Public health policies aimed at addressing these disparities - such as early screening, improved access to specialized care, and social support programs - may help reduce suffering and improve outcomes for this vulnerable population.

### Timing of Extubation

In our evaluation, there was a significant difference in the percentage of patients extubated in the operating room between the pre and post 2015 periods, 2.9% and 47.6%, respectively. Kintrup et al.^[19]^ mentioned in their study that extubation in the operating room reduced the need for antibiotics, probably due to the lower number of respiratory infections. We believe that there were individual initiatives by surgeons, anesthesiologists, and perfusionists that contributed to this significant change, as well as a gain in experience in handling the Fontan patients with the concomitant increase in the number of surgeries per year, as shown in Figure 2.

### Chest Tube Removal

In our study, the median drainage time was 6.0 (4.0; 11.0) days, which is shorter than in any of the groups reported by Mutsuga et al.^[9]^ In their study of 97 patients undergoing the Fontan procedure, three extubation strategies were compared: group A, fast-track extubation in the operating room; group B, extubation in the ICU within 24 hours; and group C, extubation after more than 24 hours. The median time to chest tube removal was 8 (4.62), 9 (2.44), and 14 (5.45) days for groups A, B, and C, respectively (*P* < 0.001). The authors concluded that fast-track extubation is feasible and associated with improved postoperative hemodynamic parameters, earlier chest tube removal, and shorter hospital stays.

Sunstrom et al.^[20]^ compared patients managed before and after the implementation of the PORTLAND protocol and found a significant reduction in drainage time (11 *vs.* 6 days, *P* < 0.001). Our results are comparable to the post-protocol group and better than the pre-protocol group.

Ovroutski et al.^[10]^ reported prolonged drainage time (> 10 days) in 40.7% of 140 patients and CPB time > 120 minutes as a risk factor for prolonged pleural effusion. In contrast, we observed prolonged drainage time in 27.1% of patients and found no association with CPB time > 120 minutes.

Mery et al.^[14]^ applied a broader definition of prolonged drainage time (> 14 days or requiring reintervention) and reported a rate of 26%. Despite our narrower definition, the proportion was similar, suggesting a relatively higher incidence. Moreover, in our cohort, 51/78 (65.4%) patients developed pleural effusion, and 23/51 (45.1%) patients required new chest tube insertion, underscoring the significant morbidity associated with this complication.

Our patients had the Fontan procedure with a mPAP of 12.7 (± 3.3) mmHg, and 22/76 (28.9%) patients had a mPAP of 15 mmHg or more; these values configure 90% specificity for unfavorable outcomes: duration of pleural effusion, prolonged hospital stay, and in-hospital death^[21]^. In fact, we observed that patients who had mPAP ≥ 15 mmHg also had a prolonged drainage time (*P* = 0.026).

Ovroutski et al.^[10]^, in a study carried out with 140 patients submitted to the Fontan procedure, identified fenestration as a risk factor for prolonged pleural effusion (longer than 10 days, with an odds ratio of 3.6 [95% CI: 1.7 - 7.8; *P* < 0.001]) and explained this finding by the fact that fenestration was performed in a subgroup with unfavorable hemodynamic factors, such as mPAP > 18 mmHg. However, in our study, there was no difference in the proportion of patients with prolonged pleural effusion between patients submitted to fenestration or not; we can conclude, therefore, that fenestration may be beneficial, but it does not necessarily need to be performed in all patients.

### Length of Stay in the Intensive Care Unit and Total Hospital Stay

In our study, the median length of stay in the ICU was 7.0 (4.0; 12.0) days, which is higher when compared to the group of patients extubated in the operating room (group A), according to the study by Mutsuga et al.^[9]^, where the median length of stay in the ICU was two (1 - 70), nine (1 - 2), and 10 (3 - 208) days respectively for groups A, B (extubation in < 24 hours), and C (prolonged extubation) (*P* < 0.001).

Mery et al.^[14]^, found a median of three (1-144) days of ICU stay in a study with 610 patients who had the Fontan procedure, even in the subgroup with patients who had acute Fontan failure (creation of fenestration, collateral occlusion, and pulmonary artery re-approach), whose median length of stay in the ICU was six (2 - 44) days.

The median length of total hospital stay found in our study was high: 24.5 (17.8; 36.5) days. Mery et al.^[14]^ reported a median of 10 (4 - 85) days of postoperative hospital stay, but in the subgroup with patients who had acute Fontan failure, the median was 27 (6 - 85) days. Ravishankar et al.^[18]^ studied patients undergoing Fontan procedure in 15 centers across North America and found a median of 11 (9 - 18) days of postoperative hospital stay.

Additionally, approximately one-fourth of the total hospital stay occurred before the surgery. This suggests that, in our institution, there is potential to reduce both pre and postoperative hospitalization times.

### Early Mortality Trend

Overall mortality in our cohort was high. However, a significant reduction in 30-day mortality was observed among patients who underwent the Fontan procedure after 2015 (4.8%) compared to those operated on before 2015 (22.9%) (*P* = 0.037), suggesting an improvement in early outcomes consistent with global trends over five decades of the Fontan procedure^[5]^. Interestingly, this improvement was accompanied by a remarkable change in perioperative practices: the rate of operating-room extubation increased approximately sixteen-fold.

This shift likely reflects a gradual improvement in perioperative care practices, which may have contributed to better early postoperative outcomes, despite the absence of a formal institutional protocol.

AKI was the strongest independent predictor of in-hospital mortality, with a multivariate HR over 65, consistent with Cardoso et al.^[22]^ who linked AKI to higher mortality in pediatric cardiac surgery, underscoring the critical impact of perioperative care.

The subgroup comparison before and after 2015 showed no difference in key baseline characteristics. However, given the small sample size, these findings should be interpreted with caution due to the potential for residual confounding.

### Hospital Readmissions

In our study, readmissions related to congenital heart disease or the Fontan procedure were caused by a variety of factors, making it difficult to establish a consistent association. The occurrence of bleeding or obstructions, pleural effusion, and arrhythmias underscores the complexity of managing these patients - including decisions regarding anticoagulation timing, optimal surgical conditions, and procedural strategies - and highlights the significant impact each of these decisions may have on reducing patient morbidity.

### Limitations

As a single-center, retrospective observational study, data were obtained exclusively from medical records, which may be incomplete or inaccurate, and information from other healthcare institutions was not systematically recorded. Given the small sample size, statistical results should be interpreted with caution due to the potential for residual confounding.

## CONCLUSION

Our study contributes to reducing the geographical gap in knowledge about the Fontan procedure, describing 78 patients who underwent Fontan procedure in South America, in a middle-income country. It shows that children who were operated on late, at the mean age of 12 years, had a long length of hospitalization and an overall mortality of 14.3%.

The findings suggest that perioperative management, particularly nutritional status, may represent a potentially modifiable risk factor.

Over time, in parallel with an increase in the number of surgeries per year, there was a decrease in early mortality and an increase in the number of patients extubated in the operating room, evidencing improved patient management.

AKI emerged as the strongest independent predictor of in-hospital mortality, highlighting the need for close perioperative monitoring and preventive strategies.

It is important to closely follow patients with functionally univentricular hearts and offer them early surgery, in order to minimize their endurance of long years of hypoxemia, with consequent higher rates of ventricular dysfunction and worse outcomes following late procedures.

Furthermore, the rate of hospital readmissions related to congenital heart disease and the Fontan procedure was high, evidencing the high morbidity suffered by these patients. Our results highlight that families of children undergoing the Fontan procedure experience socioeconomic barriers to providing adequate care.

These findings emphasize the importance of early referral, optimized perioperative care, and institutional learning in improving outcomes for Fontan patients in middle-income countries, in addition to the potential impact of public health policies in addressing these issues.

## Data Availability

The authors declare that the data will be available upon reasonable request to the authors.

## Artificial Intelligence Usage

The authors declare that no artificial intelligence tool was used in the preparation of this article.
